# Self-Assembled InAs Nanowires as Optical Reflectors

**DOI:** 10.3390/nano7110400

**Published:** 2017-11-21

**Authors:** Francesco Floris, Lucia Fornasari, Andrea Marini, Vittorio Bellani, Francesco Banfi, Stefano Roddaro, Daniele Ercolani, Mirko Rocci, Fabio Beltram, Marco Cecchini, Lucia Sorba, Francesco Rossella

**Affiliations:** 1 Dipartimento di Fisica, Università di Pavia, via Bassi 6, 27100 Pavia, Italy; francesco.flrs@gmail.com (F.F.); lucia.fornasari@unipv.it (L.F.); vittorio.bellani@unipv.it (V.B.); 2 ICFO-Institut de Ciencies Fotoniques, The Barcelona Institute of Science and Technology, 08860 Castelldefels (Barcelona), Spain; andrea.marini.tlp@gmail.com; 3 Interdisciplinary Laboratories for Advanced Materials Physics (I-LAMP) and Dipartimento di Matematica e Fisica, Università Cattolica del Sacro Cuore, Via Musei 41, 25121 Brescia, Italy; francesco.banfi@unicatt.it; 4 National Enterprise for Nanoscience and Nanotechnology (NEST), Scuola Normale Superiore and Istituto Nanoscienze-CNR, Piazza S. Silvestro 12, 56127 Pisa, Italy; stefano.roddaro@sns.it (S.R.); daniele.ercolani@sns.it (D.E.); mirko.rocci@sns.it (M.R.); fabio.beltram@sns.it (F.B.); marco.cecchini@nano.cnr.it (M.C.); lucia.sorba@nano.cnr.it (L.S.)

**Keywords:** semiconductor nanowire, nanostructured optical surface, sub-wavelength nanostructures, specular reflectance, sensing

## Abstract

Subwavelength nanostructured surfaces are realized with self-assembled vertically-aligned InAs nanowires, and their functionalities as optical reflectors are investigated. In our system, polarization-resolved specular reflectance displays strong modulations as a function of incident photon energy and angle. An effective-medium model allows one to rationalize the experimental findings in the long wavelength regime, whereas numerical simulations fully reproduce the experimental outcomes in the entire frequency range. The impact of the refractive index of the medium surrounding the nanostructure assembly on the reflectance was estimated. In view of the present results, sensing schemes compatible with microfluidic technologies and routes to innovative nanowire-based optical elements are discussed.

## 1. Introduction

New pathways for light manipulation [1] and unprecedented control of light-matter interaction at the nanoscale [2,3,4] were demonstrated exploiting the optical response of metamaterials [5,6,7], artificial media composed of repeating patterns of subwavelength elements fashioned from composite materials. In this context, metal-dielectric composite systems typically suffer from large absorption owing to Ohmic losses in the underpinning metallic building blocks, which can be detrimental for practical applications. Working in reflection geometry, nanostructured optical components overcome this limitation and offer enormous opportunities for optical manipulation [8], e.g., control of polarization [9] and orbital angular momentum of light [10], dispersive holograms [11] and spatial light modulators [12].

Dielectric systems remain however desirable at optical frequencies as they are largely free from Ohmic losses. Artificial arrangements of dielectric nanostructures including nanowires (NWs) [13] can yield engineered photonic-bandgap materials [14] and are of considerable importance for applications in next-generation light-harvesting and photovoltaics [15,16,17,18,19,20]. Silicon NWs vertically arranged and electromagnetically coupled to each other were shown to display frequency-dependent reflection spectra, enabling color matrix refractive index sensors [21]. III–V nanowire/Si triple-junction solar cells were proposed and theoretically discussed in recent years. The high absorbability of the NWs and the greatly suppressed reflection were shown to yield excellent current matching between subcells, resulting in a cell efficiency of 31.4% [22]. In this context, non-periodic semiconductor-based nano-arrangements can in turn display unique optical responses and provide intriguing photonic platforms. This was recently pointed out in experimental and theoretical studies focusing on silicon nanostructure fractal arrays and mats [23,24]. As far as concerns III–V semiconductor NW ensembles, investigation efforts were devoted to GaP-based nano-materials for photovoltaic applications, and the experimental research aimed at addressing the optical properties of the systems under test by measuring their optical transmission and birefringence [25,26,27,28].

In this work, attention was focused on the study of the optical properties of non-periodic assemblies of InAs nanowires. Here, polarization- and angle-resolved optical micro-reflectivity was investigated, and strong modulations of the polarization-resolved reflectance as a function of photon energy and incidence angle were observed. Our experimental findings were rationalized for long wavelengths in the frame of an effective-medium model and were accurately reproduced in the whole wavelength range by numerical simulations. The changes in the relative reflectance variation produced by small changes in the refractive index of the medium filling the volume between the NWs were theoretically estimated. In the present implementation, the self-assembled NW technology was exploited, which enables fast and cost-effective realization of large surfaces covered by semiconductor nanostructures. Our work provides the experimental proof-of-concept that vertically aligned InAs NW arrangements represent a promising class of nanostructured optical reflectors. Our estimations of the impact of the refractive index of the medium filling the volume between the NWs are relevant in view of sensing applications, thanks to the exceptional surface-to-volume ratio due to the high aspect ratio of the NWs.

## 2. Nanowire Assemblies and Light Reflection

InAs NW assemblies were grown on (111)B InAs substrates exploiting gold-catalyzed chemical beam epitaxy [29]. This technically gives to the samples a hybrid metal-semiconductor character, since the InAs NWs have on top Au-rich nanoparticles. Nevertheless, the Ohmic losses due to the metal could be of relevance at normal or close to normal incidence only, while at increasing incidence angle, the optical response is expected, and indeed found, to be dominated by the semiconductor. Actually, the peculiar optical features presented in this work occur at high incidence angle. In this sense, our system can be thought of as a purely semiconducting one. For this study, a subset of samples was selected, characterized by very similar average NW length (≈1 μm) and diameter (≈50 nm) and different NW densities, i.e., different substrate coverages, in the range from ≈5 NW/μm${}^{2}$ to ≈50 NW/μm${}^{2}$ (see the Supporting Information).

**Figure 1:**
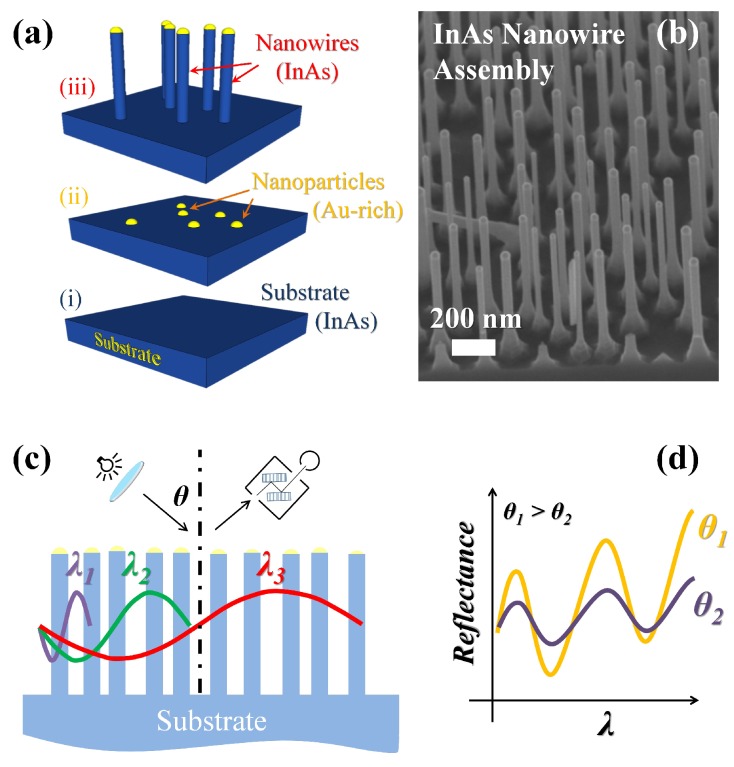
(**a**) Schematics of a set of samples used in this work: (i) the InAs substrate; (ii) the InAs substrate with dispersed Au-rich catalyst nanoparticles; (iii) the InAs substrate with the InAs nanowire (NW) assembly; (**b**) ≈ 45${}^{\circ }$ tilted scanning electron micrograph of InAs NWs (density ≈ 30 NW/μm${}^{2}$, diameter ≈ 50 nm, length ≈ 950 nm); (**c**) as light impinges on the NW assembly, photons with a short wavelength interact with few individual NWs, while large wavelengths see the whole assembly as an effective-medium; (**d**) diffraction and interference effects give rise to reflectance oscillations versus photon wavelength and incidence angle.

As mentioned above, the semiconductor nanostructures can in principle compete with the Au-rich NW tips (Figure 1a(iii),b) in determining the optical response of the assembly, in particular since the metallic nanoparticles may support surface plasmon resonances. However, the latter are known to be strongly dependent on the specific photon incident wavelength and angle and are thus relatively easy to identify. In the present case, as will be shown further on, significant features ascribable to the metallic tips were not observed, neither in the experimental reflectance spectra, nor in the calculated ones. This can be due to the small cross-section of the metallic tip, rapidly becoming negligible with respect to that of the whole semiconductor volume of the NW for increasing incidence angle and to the chemical composition of the catalyst, where the indium amount can reach ≈35%. Sets of three types of samples were investigated (see Figure 1a): the bare InAs substrate, the substrate with the dispersed Au-rich nanoparticles (NPs) and the substrate with the InAs NWs. For different substrate coverages, it was verified that the density of the NPs equals the density of the NWs. The Fourier analysis of binarized top-view scanning electron microscopy (SEM) micrographs of the samples returns spot-less spectra, consistent with the absence of long-range order in the NW motif. Details on the growth and the morphological characterization of the samples are reported in the Methods and the Supporting Information.

As white light impinges on the NW assembly with a characteristic NW-to-NW distance, photons can either interact with individual NWs and undergo multiple scattering within the NWs (short wavelengths) or experience the effective optical response of the system (long wavelengths), as schematically illustrated in Figure 1c. This yields interference effects (due to relative phase difference between reflected waves) that in general depend on the incident photon wavelength, thus affecting the far-field response that may display the appearance of peculiar features (such as oscillations) in the spectrally-resolved reflectance of the assembly (Figure 1d). Reflectance modulations not strictly dependent on the geometry of the arrangement make the optical response particularly robust and stable and protect it from fluctuations of the NW-to-NW distance. This, together with the very large surface-to-volume ratio, due to the high aspect ratio of the NWs, makes the present system very attractive for sensing applications.

## 3. Experimental Results: Optical Reflectance

**Figure 2:**
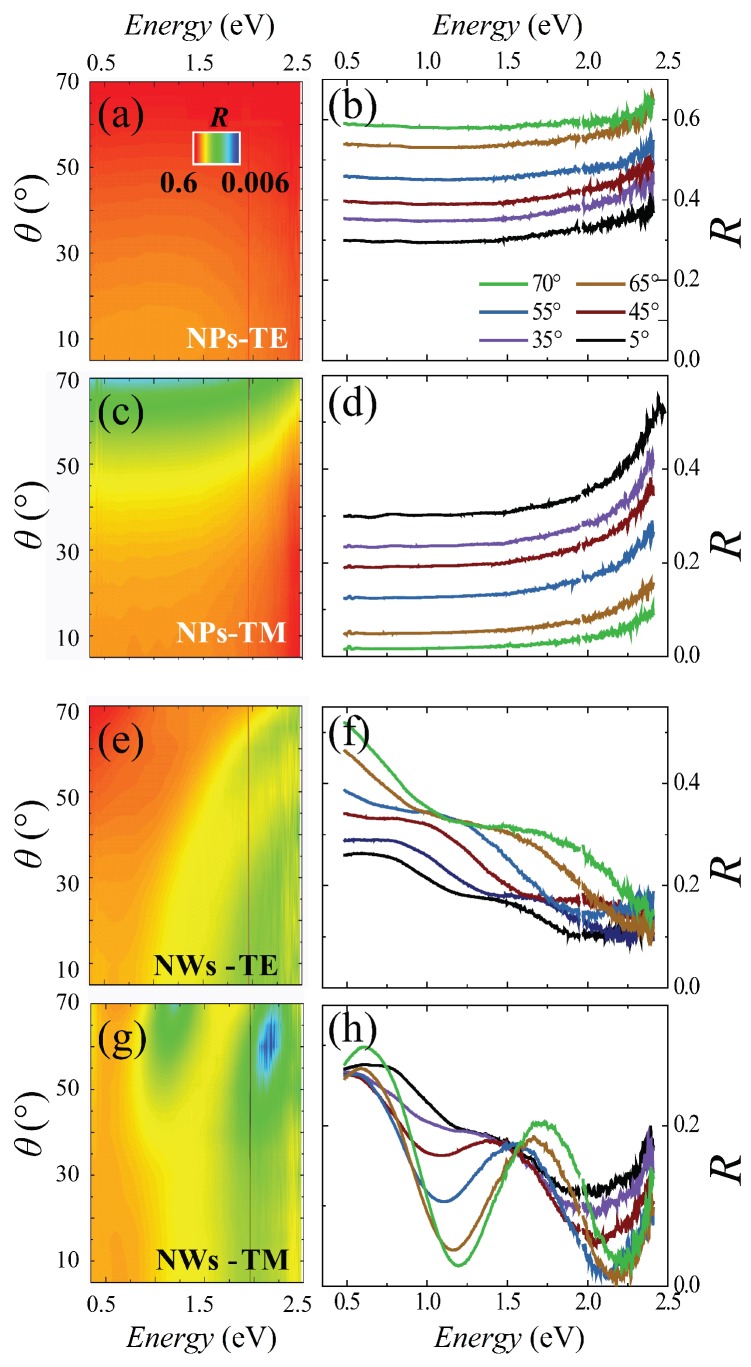
Angle- and energy-resolved reflectance modulations. Logarithmic color plot of the angle- and energy-resolved specular reflectance measured on the InAs substrate with the dispersed Au-rich NPs (**a**,**c**) and on the InAs NWs (**e**,**g**). Reflectance spectra measured at different incident photon angle θ (indicated by different color curves) for the Au-rich NPs dispersed on the InAs substrate (**b**,**d**) and for the InAs NWs (**f**,**h**). Transverse electric (TE) and magnetic (TM) light polarizations are indicated by labels. Marked reflectance modulations versus photon energy and incidence light angle occur in the InAs NWs, while almost featureless reflectance is observed for the InAs substrate with dispersed Au-rich NPs.

In order to probe the capability of InAs NW assemblies to manipulate an impinging electromagnetic field, we experimentally investigate the far-field optical response by angle-resolved specular reflectance (*R*) measurements [14,30]. Figure 2 shows *R* for the case of an InAs NW sample with ≈30 NW/μm${}^{2}$ (Panels e–h) and for the substrate with the dispersed NPs only (Panels a–d). *R* was measured for transverse electric (TE) and magnetic (TM) polarized light covering the visible and near-IR frequency range, as a function of the photon incidence angle θ (between 5${}^{\circ }$ and 70${}^{\circ }$). The spectra of the bare substrate (not shown) and of the substrate with dispersed NPs display almost identical features, indicating that the Au-rich NPs dispersed on the substrate do not play a significant role in the explored range of photon energy and incidence angle. TE and TM reflectance plots for the InAs substrate with the Au NPs are reported in Figure 2a,c in a logarithmic color scale as a function of photon energy and incidence angle. They show a monotonic behavior both in the angle- and energy-dependence of the reflectance. The cross-cuts at different photon angles reported in Figure 2b,d reveal almost flat, featureless spectra for both polarizations. The reflectance increases (decreases) at increasing photon incident angle for TE (TM) polarization and eventually vanishes as θ approaches the Brewster angle ($\theta_{B}\approx$ 75${}^{\circ }$) for TM-polarized light. Similar featureless spectra were measured also for low-density InAs NW samples with substrate coverage ≤10 NW/μm${}^{2}$ (see Supporting Information). Figure 2e,g shows the color-plot of the reflectance for a high-substrate coverage (≈30 NW/μm${}^{2}$) InAs NWs sample, while the spectra corresponding to crosscuts at different angles are shown in Figure 2f,h. For the TE case, even though the general increase of reflectance as a function of θ qualitatively mimics the behavior observed on the substrate, the spectra reveal an evident monotonic decrease of *R* with photon energy, with marked changes in the slope within each spectrum and between spectra measured at different angles. For the TM case, the InAs NWs display a strong oscillating optical response, as highlighted in Figure 2g,h: the color plot and the spectra are drastically different from those measured on the InAs substrate with dispersed NPs (see Figure 2c,d). In fact, the TM reflectance for the InAs NWs displays marked oscillations as a function of photon energy at different θ.

**Figure 3:**
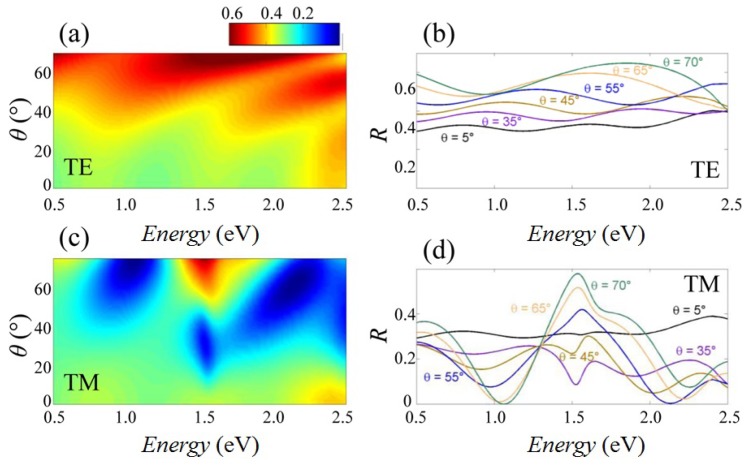
Effective-medium calculation of reflectance modulations. Logarithmic color plot of the angle- and energy-resolved reflectance calculated for InAs NWs using the effective-medium approximation for (**a**) TE polarization and (**c**) TM polarization, respectively. Reflectance spectra calculated at several different angles, for (**b**) TE and (**d**) TM polarizations.

**Figure 4:**
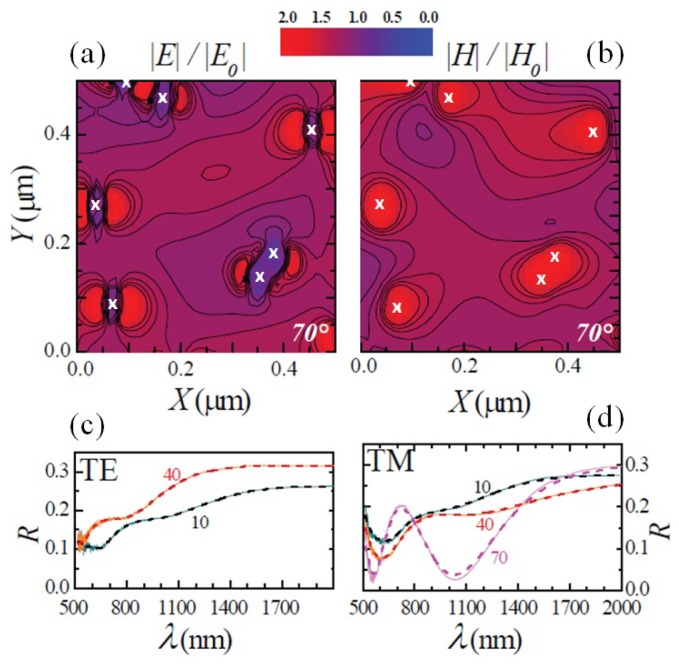
Numerical simulations of the near-field spatial distributions and reflectance. Normalized spatial distribution of the electric (**a**) and magnetic (**b**) field, calculated for the InAs NWs (density ≈ 30 NW/μm${}^{2}$, length 950 nm, diameter ≈ 45 nm) for TM-wave at 70${}^{\circ }$ and at λ≈ 1035 nm. The white crosses indicate the centers of the NWs. $E_{0}$ and $H_{0}$ represent the incident electric and magnetic field, respectively. (**c**,**d**) Calculated (dashed line) and measured (solid line) reflectance for the two polarizations.

In particular, the reflectance vanishes for θ≈ 60${}^{\circ }$ at ≈2.1 eV (≈590 nm), indicating resonant transmission of the assembly followed by absorption in the substrate for this impinging photon energy and angle. TE reflectance modulations are less strong with respect to the TM ones, as evidenced in Figure 2 and also confirmed by the simulations reported further ahead (Figure 3 and Figure 4). This behavior can be ascribed to the strong anisotropy of the NWs [31] and to a more advantageous coupling between the TM field and the lateral surface of the NWs. In fact, increasing the incidence angle from 0${}^{\circ }$ to 70${}^{\circ }$, for TE waves, the direction of the electric field is unchanged, and the cross-sections between the electric field and the NWs are small; while for TM waves, the direction of the electric field changes, and its component along the NW axis increases significantly. Further analysis of the observed trends for the InAs NWs (e.g., dependence of the intensity and energy position of peaks and valleys upon the incidence angle) is reported in the Supporting Information.

## 4. Theoretical Results: Analytical and Numerical Simulations

An effective medium model was adopted to account for the observed phenomenology and to catch the physics of the reflectance behavior in the subwavelength reflector. We assumed a medium composed of InAs cylinders with surface density $N_{C}$ and radius *r* much smaller than the optical wavelength λ. In this limit, the composite medium behaves like a material with effective dielectric constants given by the Maxwell–Garnett average over the transverse plane perpendicular to the NW growth direction, $\epsilon_{⊥,InAs}$, and the arithmetic average over the wires axis, $\epsilon_{z,InAs}$ [32]:

(1) $$\epsilon_{⊥,InAs}=\left[pf_{C}\epsilon_{C}+\left(1-p\right)\epsilon_{B}\right]/\left[pf_{C}+\left(1-p\right)\right]\;,$$

(2) $$\epsilon_{z,InAs}=p\epsilon_{C}+\left(1-p\right)\epsilon_{B}\;,$$

where $p=\pi^{2}r^{2}N_{C}$ is the cylinder medium filling factor (i.e., areal NW packing fraction), $\epsilon_{C}$ is the dielectric constant of the cylinder medium, $\epsilon_{B}$ is the dielectric constant of the background medium and $f_{C}$= 2$\epsilon_{B}$/($\epsilon_{C}$ + $\epsilon_{B}$). For the sake of completeness, the metallic nanoparticles on top of the nanowires have been taken into account, making a similar average to achieve the effective dielectric constants $\epsilon_{⊥,Au}$ and $\epsilon_{z,Au}$. In order to calculate optical propagation, we assumed monochromatic waves with angular frequency ω and wavevector ***k*** = ($k_{x}$, $k_{z}$) impinging on the effective-medium described above. The full analytical solutions of Maxwell’s equations (Supporting Information) can be split into two sub-sets of solutions for TE- and TM-polarized waves, and the results are reported in Figure 3. The dependence of the calculated reflectance on photon incidence angle and energy reproduces the main experimental trends, and in general, our model better reproduces the experimental features at smaller incident photon energy. This occurs since the assumptions of the effective-medium approximation ceases to hold as the wavelength becomes shorter and thus light probes individual NWs rather than the NW assembly as a whole. Some minor differences between experimental and calculated curves are observed: for TM polarization, in the range 20${}^{\circ }$ ≤ θ ≤ 40${}^{\circ }$, a dip appears at ℏω≈ 1.5 eV; the measured data display, superimposed on the oscillations, a general decrease of *R* at increasing photon energy, which is not observed in the theoretical curves; calculated peaks and dips of *R* are slightly blue-shifted with respect to the measured ones; the experimental reflectance is quenched with respect to the theoretical one.

In order to fully recover the fine details of the experimental curves over the whole range of energy and angles, Maxwell’s equations were solved numerically by resorting to a finite-difference time-domain (FDTD) code [33,34] in a model system composed of a quasi-random motif of vertically-aligned, identical NWs with diameters, lengths and densities mimicking the investigated InAs NWs. This allowed us to address the far- and near-field response of our system. Figure 4a,b reports the electric and magnetic near-field spatial distributions for the InAs NWs. Our calculations show a significant electric field at the NW lateral surfaces (near field) and confirm the occurrence of marked resonances in the reflectance (far field): the light scattered within and reflected from the NW surface displays a large modulation that depends on the polarization, wavelength and incidence angle of the radiation. Panels c and d in Figure 4 show the reflectance spectra calculated for InAs NWs at different θ (dashed curves) together with the corresponding experimental spectra (solid curves): the agreement is remarkable in the whole wavelength range examined. The longitudinal electric field expansion was also simulated for different angles of incidence, and the results can be found in the Supporting Information.

## 5. Discussion: Sensing Applications

**Figure 5:**
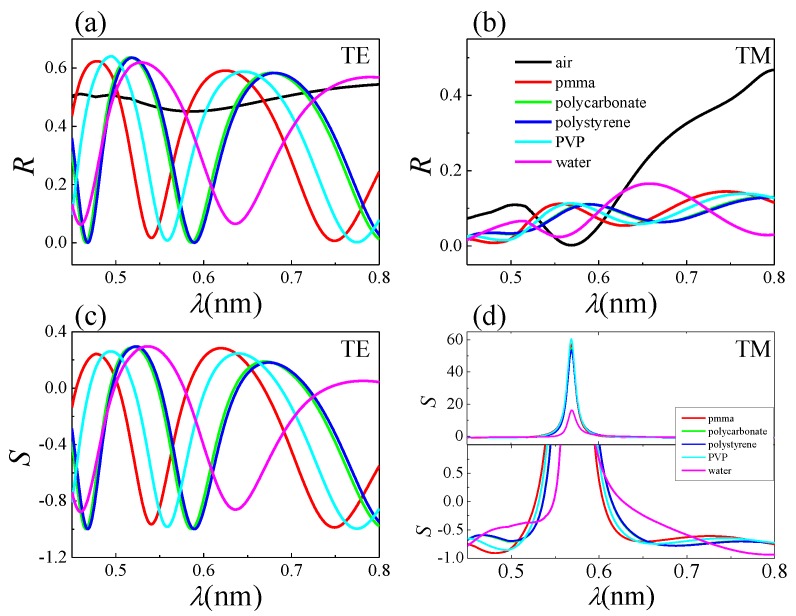
Effect of different media filling the InAs nanowire assembly. Optical response calculated for different media surrounding the NWs, for TE (**a**) and TM (**b**) polarization. The filling media are indicated in (**b**). Sensitivity or relative reflectance variation *S* = (*R* − $R_{\mathrm{air}}$)/$R_{\mathrm{air}}$ for TE (**c**) and TM (**d**) polarizations. The incidence angle is 55${}^{\circ }$.

Owing to their large surface-to-volume ratio, NWs and NW-based systems bear great potential for sensing applications. For instance, semiconductor nanowire field-effect sensor devices were proposed as a powerful detection platform for a broad range of biological and chemical species in solution [35]. Here, the attention is focused on a different, all-optical sensing paradigm. To this end, the optical response of the NW assembly was analytically estimated for different filling media (i.e., different refractive indexes in the space surrounding the NWs), in the frame of the effective model presented above. A similar approach was recently used for the theoretical estimation of the influence of wetting states on the reflectance of Si nanopillars [36,37]. As the filling medium, besides air, we considered water in light of its relevance for biosensing applications. We also considered transparent thermoplastics (polymethyl methacrylate (PMMA) and polycarbonate) and synthetic aromatic polymers (polystyrene and polyvinylphenol or polyvinylpyrrolidone (PVP)), commonly used in the science and technology of semiconductors. Panels a and b in Figure 5 show the reflectance versus wavelength at fixed incidence angle in the case of air, water and four different polymers, for the TE and TM case, respectively. Panels c and d in Figure 5 show the sensitivity *S* calculated as the relative reflectance variation, i.e., *S* = (*R* − $R_{air}$)/$R_{air}$. In these calculations, an incident angle θ = 55${}^{\circ }$ at which a set of reflectance resonances is observed was chosen. We notice that TE modulations may become relevant and even stronger with respect to the TM ones. In fact, the presence of a medium with permittivity higher than air surrounding the NWs yields a longer effective optical path, affecting interference (and diffraction) phenomena and thus reflectance modulations. From the present calculations, it can be estimated that, over the whole photon energy range, a variation △n ≈ 0.01 of the refractive index *n* of the filling medium causes a change up to △R ≈ 0.05 of the reflectance, which is well above the present detection limit, as shown by the experimental curves in Figure 2. In particular, assuming water as the medium filling the NW reflector, for △n ≈ 0.01, we get △R ≈ 0.01, which appears compatible with the present experimental arrangement.

An experimental demonstration of sensing functionalities in our system goes beyond the purpose of the present work. However, the present investigation can be of relevance for the development of all-optical cost-effective NW-based sensing platforms compatible with microfluidic integration [38,39], where the sensing area of the chip is made of NWs, light is focused at a fixed incidence angle and the reflectance versus wavelength is probed. For instance, when a small quantity of fluid is driven within a microfluidic circuit into the NW-based reflector (with a size scalable down to a few micrometers), the liquid fills the gaps around the NWs, and the reflectance spectrum changes. A straightforward application would be the measurement of the liquid refractive index by comparing the experimental curve against a calibration library. The present platform enables label-free detection and does not involve surface plasmon resonance and complex system modeling. Furthermore, we believe that an important application would be the detection of a specific biological-complex formation [40,41]. To this aim, for different specific binding events considered, different biorecognition elements should be used to functionalize the NW surfaces. The analyte molecules present in the solution spread between the NWs, bind to the biorecognition elements and cause an increase in the refractive index *n* at the NW surface. The change of *n* produced by the binding event depends on the properties of the molecules and on the concentration of the analyte molecules at the NW surface. If the binding occurs within a thin layer at the NW surface, the sensor response will be proportional to the binding-induced *n* change and ultimately to the analyte surface concentration, i.e., the ratio between the analyte-molecule mass and the NW area. In this scenario, the high surface-to-volume ratio characteristic of NWs can play a very favorable role. Finally, it is worth noting that the present sensing strategy, based on disordered disposition of nanowires, is recently being pursued also in other frames such as that of the granular nanoparticle system for gas and liquid sensing applications [42].

Here, starting from quasi-randomly textured NW assemblies characterized by a relatively broad distribution of diameter and, to a lesser extent, length, we have shown that non-trivial optical response (reflectance) can be achieved for a suitable average areal NW packing fraction and that the observed phenomenon could be exploited for sensing applications. With a different approach, the flexibility of the epitaxial-growth technique (in terms of materials, heterostructure architectures and dimensions) could be combined with the accurate control of NW morphology and location (achievable using electron beam lithography to define the arrangement of the Au catalyst nanoparticles). These, together with a robust modeling effort yielding predictive input for the NW array design, could be the key ingredients for the realization of fully-engineered all-dielectric NW-based optical elements and metasurfaces, opening the way toward exciting opportunities for light manipulation at the nanoscale.

## 6. Methods

InAs NWs were grown by Au-assisted chemical beam epitaxy in a Riber Compact 21 system, employing pressure control in the metalorganic lines to determine precursor fluxes during the growth. The precursors involved in the NW growth are tri-methylindium (TMIn) and tertiarybutylarsine (TBAs). A nominally 0.5 nm-thick Au film was first deposited on (111)B InAs wafers by thermal evaporation. Before the growth was initiated, the sample was heated at $470\pm 10{\;}^{\circ }C$ under TBAs flow for 20 min in order to de-wet the Au film into nanoparticles and to remove the surface oxide from the InAs substrate. The NWs were grown at a temperature of ≈385 ± 10 ${}^{\circ }$C, with TMIn and TBAs flux of ≈0.3 Torr and ≈0.7 Torr, respectively, for a time of ≈45 min.

Variable-angle specular reflectance was measured over the spectral range from 2000 to 20,000 cm${}^{-1}$ (0.248–2.479 eV). The light of a Xe arc-lamp was collimated and then focused on the sample into a spot having a diameter of 1–100 μm and divergence less than 2${}^{\circ }$. A home-made micro-reflectometer setup associated with a Fourier transform spectrophotometer Bruker IFS66 (spectral resolution of 0.15 cm${}^{-1}$) was used to investigate the samples. For the dispersion measurements, the in-house built goniometer allows varying the incidence angle θ between 5${}^{\circ }$ and 70${}^{\circ }$, while the reflected beam was collected at 2θ. A Glan Taylor polarizer was used to select TE (*s*) or TM (*p*) polarized light. In order to measure the reflectance, two silicon and an InSb photodiodes (with detectivity extending from 400 nm to 1200 nm and from 1000 nm to 5000 nm, respectively) were used.

Finite-difference time-domain (FDTD) numerical simulations of the electric field distributions and of the optical reflectance spectra were performed using Lumerical FDTD Solutions software. The bare nanostructures were modeled as bulk substrates overlaid with an assembly of vertically-aligned nanowires. In our code, we used five randomly-displaced deterministic non-periodic arrays that mimic the non-periodic and non-deterministic NW texture. The result is a good approximation, for our purpose, of the NWs assemblies used in the present experiments. The electromagnetic field (ELM) field was calculated in several planes parallel to the xy one at different values of *z*.

## 7. Conclusions

In conclusion, the optical reflection in assemblies of vertically-aligned InAs nanowires was investigated. Marked reflectance oscillations were achieved that are not critically driven by the detailed arrangement of the nano-objects, but instead stem from a suitable choice of substrate filling factor (or areal NW packing fraction), a macroscopic property, together with the refractive-index contrast occurring at the nanoscale. Theoretical modeling, which provides a nice agreement with the experiments, strongly supports this viewpoint. In fact, an effective-medium model and numerical simulations allow one to rationalize and accurately reproduce the experimental findings in the whole energy range examined. The large surface-to-volume ratio assured by the characteristic aspect ratio of the NWs is a very desirable feature for sensing applications and may pave the way toward a new class of robust, semiconductor technology-compatible, cost-effective, all-optical nanoscale sensors. Change C4: The bio-sensing paradigm here presented can be summarized as follows. The biomolecules to be detected are dispersed in the liquid, which infiltrates in the InAs nanowire forest. When these target molecules selectively anchor to the probes that were previously immobilized on the nanowire surface, a change in the optical constants occurs, which in turn modifies the optical response of the infiltrated NW forest.

## Supplementary Materials

The following are available online at www.mdpi.com/2079-4991/7/11/400/s1. Details on the growth and characterization of the samples. Reflectance data for the low-substrate coverage NW sample. Energy dispersion of the reflectance minima for InAs NWs. Full analytical solutions of Maxwell’s equations for the effective-medium model.
