# Supplementary material for: Self-Assembled InAs Nanowires as Optical Reflectors
Source: Nanomaterials (Basel). 2017 Nov 21;7(11):400. doi: 10.3390/nano7110400 (PMC5707617; doi:10.3390/nano7110400)
Supplement: Supplementary file 1 [file nanomaterials-07-00400-s001.pdf]

Self-assembled InAs nanowires as optical reflectors

Francesco Floris [1], Andrea Marini [2], Lucia Fornasari [1], Vittorio Bellani [1], Francesco Banf [3], Stefano Roddaro[4], Daniele Ercolani [4], Mirko Rocci [4], Fabio Beltram [4], Marco Cecchini [4], Lucia Sorba [4] and Francesco Rossella [4,*]

1 Dipartimento di Fisica, Università di Pavia, via Bassi 6, 27100 Pavia, Italy
2 ICFO-Institut de Ciencies Fotoniques, The Barcelona Institute of Science and Technology, 0886 , Castelldefels (Barcelona), Spain
3 Interdisciplinary Laboratories for Advanced Materials Physics (I-LAMP) and Dipartimento di Matematica e Fisica, Università Cattolica del Sacro Cuore, Via Musei 41, 25121 Brescia, Italy
4 NEST, Scuola Normale Superiore and Istituto Nanoscienze-CNR, Piazza S. Silvestro 12, I-56127 Pisa, Italy

Supporting Information

1. InAs nanowire samples and morphological characterization

Exploiting the chemical beam epitaxy technique, we have grown gold catalyzed InAs nanowire (NW) samples characterized by different average length (from 250-to 2000 nm), average diameter (from 25 to 60 nm) and NW density (from ≈ 5 to ≈ 50 NWs/μm^2). Each sample was imaged using scanning electron microscopy (SEM), and the images were processed using a in house developed Mathematica code [Gomes, U.P. et al, *Semicond. Sci. Technol.* 30, 115012 (2015)]. that performs automatic morphological analysis of a statistical population of individual NWs (> 1000). Typically, standard deviations for NW diameter and length were ≈ 8 nm and ≈100 nm, respectively.

Among the samples analyzed, those with average length of ≈ 1 μm and average diameter of ≈ 50 nm displayed high NW homogeneity and narrow distributions, and allowed to preserve these characteristics for different coverage of the substrate with NWs, from ≈ 5 NWs/μm^2 to ≈ 50 NWs/μm^2.

As an example, in Figs. SI.1 a-c we report 45° tilted SEM micrographs of three InAs NW assemblies (samples A, B and C) characterized by about the same NW average length (≈ 1 μm) and NW average diameter (≈ 50 nm) but different substrate coverage.

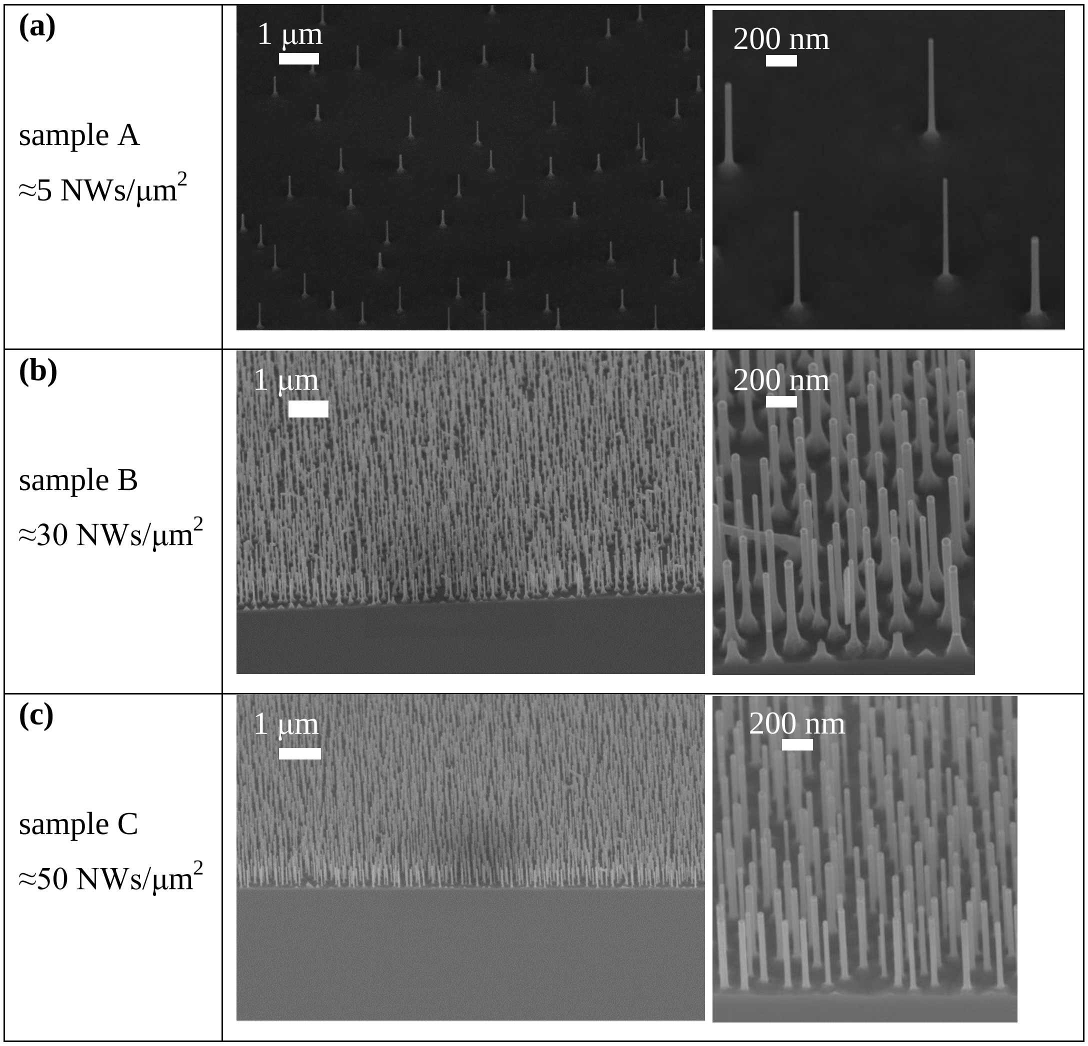

Figure SI.1. (a-c) 45° tilted SEM micrographs of InAs NW samples with similar NW average dimensions (≈ 1 μm length and ≈ 50 nm diameter) but different substrate coverage, as indicated.

We carried out the 2D Fourier analysis of several top-view SEM micrographs of each samples taken with different magnifications. This gave us an indication of the random character of the NW arrangements. This is evident in Fig.SI.2, where we report SEM images (quasi top-view) of sample B. Binarized images such as the one shown in panel (c) were processed using the FFT tool of free image processing software (ImageJ). The results - see the plot shown in panel (d) - confirm the absence of long range order in the NW arrangement and likely reflect the average shape (hexagonal) of the NW cross-section.

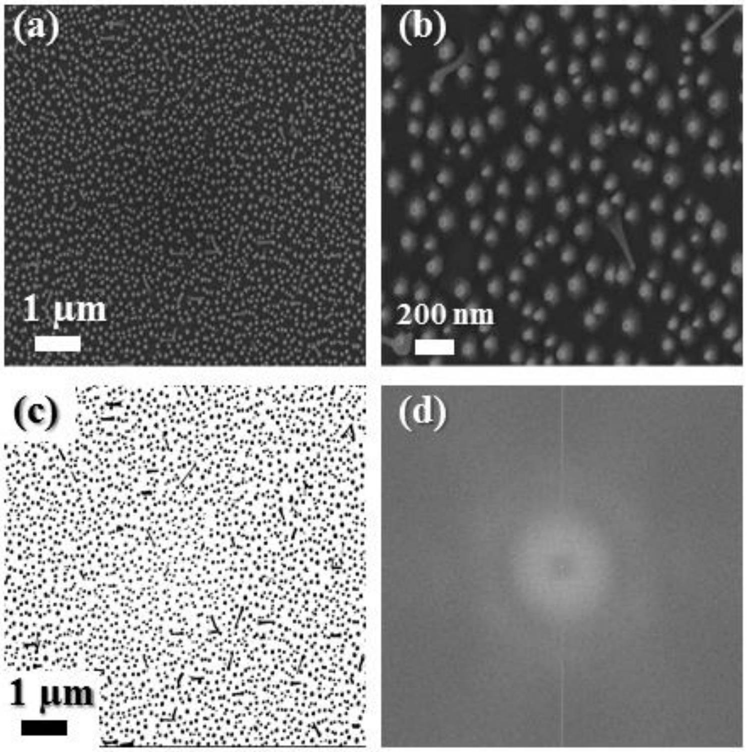

Figure SI.2. (a,b) quasi top-view SEM micrographs of InAs NW sample B; **(c)** the same SEM micrograph as in (a) after being binarized. **(d)** fast Fourier transform (FFT) of the image reported in (c). The absence of dot patterns accounts for the disordered NW arrangement.

2. Optical response in low-substrate coverage InAs NWs sample

We have observed non-negligible features in the reflectance spectra only for NW samples characterized by substrate coverage well exceeding 10 NWs/μm^2. For samples with $\leq$ 10 NWs/μm^2, the reflectance spectra were practically indistinguishable from the spectra measured for the bulk substrate or for the substrate covered with catalyst nanoparticles (with NPs/μm^2 $\approx$ NWs/μm^2). As an example in Fig. SI.3 we compare the reflectance spectra (both transverse-electric, TE, and transverse-magnetic, TM) measured for the sample A (see Fig. SI.1a), characterized by a substrate coverage of $\approx$5 NWs/μm^2, and the spectra measured for the substrate covered with catalyst NPs.

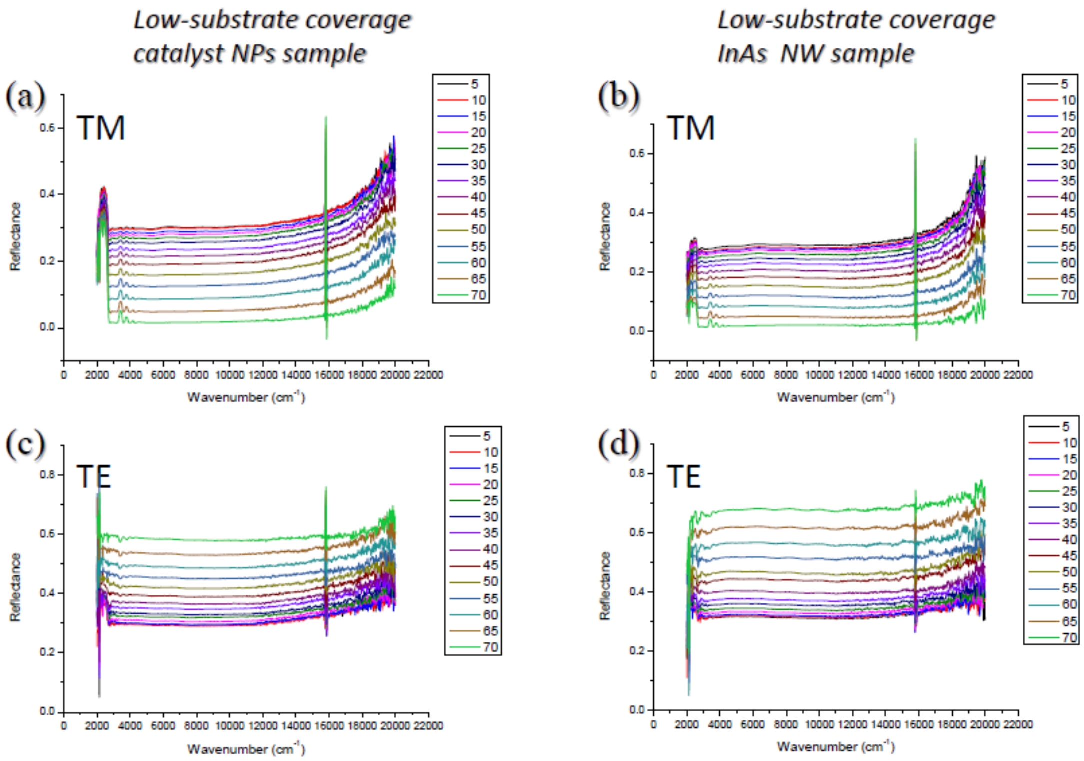

Figure SI.3. Specular reflectance spectra measured for TE and TM configuration as function of the incidence angle varying between 5° and 70°, for a low-substrate coverage g InAs NW sample and for a substrate simply covered with NPs: **(a)** substrate with NPs, TM configuration; **(b)** same configuration for InAs NW sample; **(c)** substrate with NPs, TE configuration; **(d)** same configuration for InAs NW sample.

3. Angular dispersion of the intensity and energy of the TM reflectance minima

Reflectance spectra were measured in the InAs NW assembly for TM light polarization, increasing the incidence angle from 5° to 70°. The experimental curves display a minimum at about 1 eV (Min-1) and a minimum at about 2 eV (Min-2). We probed a marked change of the intensity and energy position of these minima, as reported in Fig. SI.4 In particular, blue-shift of ~0.1 eV and ~0.16 eV for Min-1 and Min-2 were detected, respectively. The peak-to-valley ratio evolves with θ as well, increasing for both minima.

.

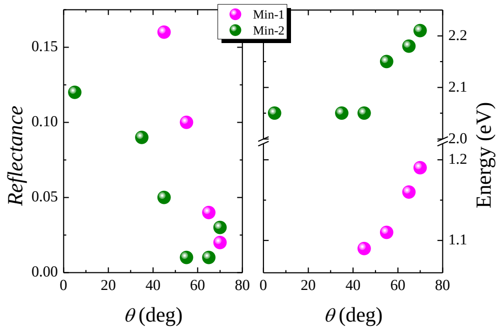

Figure SI. 4. Angular dispersion of the minima observed in TM polarization. (a) Intensity of the reflectance at Min-1 and MIN-2. **(b)** Energy position of Min-1 and Min-2.

In addition, the longitudinal near-field distribution at different angle of incidence (0°, 55° and 70°) was simulated resorting to the FDTD code described in the manuscript. The resulting local electric field shape changes are reported in Fig. SI.5. The outcomes of the calculation is consistent with the experimental trends reported in Fig. 2 of the manuscript. In fact, the increase of the angle of incidence involves a strengthening of the local field intensity associated to the formation of a dip in the reflection. In addition, it is evident the confinement of the field mainly at the upper surface of the NWs, that can represent a useful feature for sensing application.

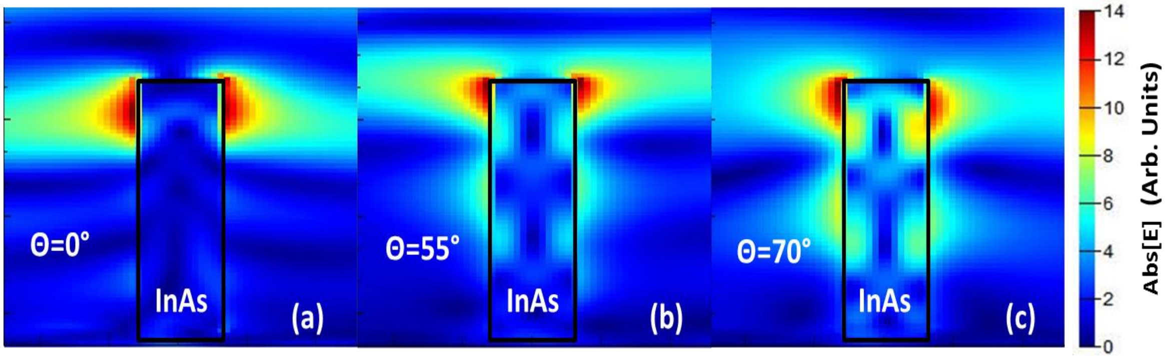

Figure SI.5. Longitudinal electric field intensity distribution around a NW with respect to the angle of incidence (θ) in TM polarization for the Min-1. (a) θ=0°. **(b)** θ=55°. **(c)** θ=70°.

It's worth noting that, placing an appropriate gold coating at the top of the nanowires i.e. in the region where the field is maximum, the nanostructures could become even more suitable for sensing applications. In Fig. SI.6 we report the results for a 30 nm thick Au-coating. The latter is found to yield a significant enhancement of the electric field in the proximity of the nanowire-Au contact area, that can be likely ascribed to localized plasmon effects.

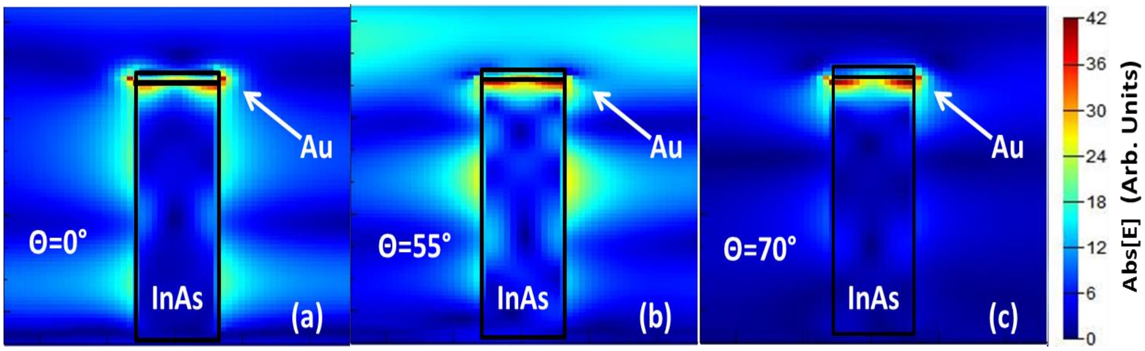

Figure SI.6. Electric field intensity distribution with respect to the angle of incidence (θ) in TM polarization for a gold coated structure. (a) θ=0°. **(b)** θ=55°. **(c)** θ=70°.

4. Effective–medium model for the InAs NWs assembly: optical propagation

We assume monochromatic waves with angular frequency ω and wavevector $\mathbf{k} = k_x\hat{x} + k_z\hat{z}$ impinging on the effective multi-layer described in the main text. Maxwell's equations provide:

$$
\begin{aligned}
&i\mathbf{k}\cdot\vec{\vec{\varepsilon}}\,\mathbf{E} = 0\\
&i\mathbf{k}\times\mathbf{E} = i\omega\mu_0\mathbf{H}\\
&i\mathbf{k}\times\mathbf{H} = -i\omega\varepsilon_0\vec{\vec{\varepsilon}}\,\mathbf{E}
\end{aligned}
$$

Thus, the problem can be split into two sub-problems for TE and TM-polarized waves. For the sake of compactness, we rename the effective dielectric constants of the layer of gold nanoparticles (with radius a) and of the underlying nanowire assembly of length l as $\varepsilon_{\perp 1,}\varepsilon_{z1}$ and $\varepsilon_{\perp 2,}\varepsilon_{z2}$, respectively. The dielectric constant of the bulk substrate is indicated as ε_S. Reflectance properties of the multi-layer medium are studied below as a function of the incidence angle $\theta_i = \mathrm{atan}\left(k_x/k_z\right)$ of the impinging optical beam.

<u>TE polarization</u>. We first assume a TE polarized field $\left(E_x = E_z = 0\right)$:

$$
\left.
\begin{aligned}
&\mathbf{E}(x,z,t) = \left[E_I e^{i\frac{\omega}{c}\cos\theta_i z} + E_R e^{-i\frac{\omega}{c}\cos\theta_i z}\right] e^{i\frac{\omega}{c}\sin\theta_i x - i\omega t}\,\hat{y}\\
&\mathbf{H}(x,z,t) = \frac{1}{\mu_0 c}\left\{\cos\theta_i\left[E_R e^{-i\frac{\omega}{c}\cos\theta_i z} - E_I e^{i\frac{\omega}{c}\cos\theta_i z}\right]\hat{x} + \sin\theta_i\left[E_I e^{i\frac{\omega}{c}\cos\theta_i z} + E_R e^{-i\frac{\omega}{c}\cos\theta_i z}\right]\hat{z}\right\} e^{i\frac{\omega}{c}\sin\theta_i x - i\omega t}
\end{aligned}
\right\} \quad z<0
$$

$$
\left.
\begin{aligned}
&\mathbf{E}(x,z,t) = \left[E_{1+} e^{i\frac{\omega}{c}\sqrt{\varepsilon_{\perp 1}-\sin^2\theta_i}\,z} + E_{1-} e^{-i\frac{\omega}{c}\sqrt{\varepsilon_{\perp 1}-\sin^2\theta_i}\,z}\right] e^{i\frac{\omega}{c}\sin\theta_i x - i\omega t}\,\hat{y}\\
&\mathbf{H}(x,z,t) = \frac{1}{\mu_0 c}\left\{\sqrt{\varepsilon_{\perp 1}-\sin^2\theta_i}\left[E_{1-} e^{-i\frac{\omega}{c}\sqrt{\varepsilon_{\perp 1}-\sin^2\theta_i}\,z} - E_{1+} e^{i\frac{\omega}{c}\sqrt{\varepsilon_{\perp 1}-\sin^2\theta_i}\,z}\right]\hat{x} + \sin\theta_i\left[E_{1+} e^{i\frac{\omega}{c}\sqrt{\varepsilon_{\perp 1}-\sin^2\theta_i}\,z} + E_{1-} e^{-i\frac{\omega}{c}\sqrt{\varepsilon_{\perp 1}-\sin^2\theta_i}\,z}\right]\hat{z}\right\} e^{i\frac{\omega}{c}\sin\theta_i x - i\omega t}
\end{aligned}
\right\} \quad 0<z<a
$$

$$
\left.
\begin{aligned}
&\mathbf{E}(x,z,t) = \left[E_{2+} e^{i\frac{\omega}{c}\sqrt{\varepsilon_{\perp 2}-\sin^2\theta_i}\,z} + E_{2-} e^{-i\frac{\omega}{c}\sqrt{\varepsilon_{\perp 2}-\sin^2\theta_i}\,z}\right] e^{i\frac{\omega}{c}\sin\theta_i x - i\omega t}\,\hat{y}\\
&\mathbf{H}(x,z,t) = \frac{1}{\mu_0 c}\left\{\sqrt{\varepsilon_{\perp 2}-\sin^2\theta_i}\left[E_{2-} e^{-i\frac{\omega}{c}\sqrt{\varepsilon_{\perp 2}-\sin^2\theta_i}\,z} - E_{2+} e^{i\frac{\omega}{c}\sqrt{\varepsilon_{\perp 2}-\sin^2\theta_i}\,z}\right]\hat{x} + \sin\theta_i\left[E_{2+} e^{i\frac{\omega}{c}\sqrt{\varepsilon_{\perp 2}-\sin^2\theta_i}\,z} + E_{2-} e^{-i\frac{\omega}{c}\sqrt{\varepsilon_{\perp 2}-\sin^2\theta_i}\,z}\right]\hat{z}\right\} e^{i\frac{\omega}{c}\sin\theta_i x - i\omega t}
\end{aligned}
\right\} \quad a<z<l
$$

$$
\left.
\begin{aligned}
&\mathbf{E}(x,z,t) = E_S e^{i\frac{\omega}{c}\sin\theta_i x + i\frac{\omega}{c}\sqrt{\varepsilon_S-\sin^2\theta_i}\,z - i\omega t}\,\hat{y}\\
&\mathbf{H}(x,z,t) = \frac{1}{\mu_0 c}\left[\sin\theta_i\hat{z} - \sqrt{\varepsilon_S-\sin^2\theta_i}\,\hat{x}\right] E_S e^{i\frac{\omega}{c}\sin\theta_i x + i\frac{\omega}{c}\sqrt{\varepsilon_S-\sin^2\theta_i}\,z - i\omega t}
\end{aligned}
\right\} \quad z>l
$$

Imposing boundary conditions for the continuity of the magnetic field and the tangential electric field at all the interfaces one gets $\boxed{M\mathbf{e}=\mathbf{b}}$ where:

$$q_1=\sqrt{\varepsilon_{\perp 1}-\sin^2\theta_i}\,,\qquad q_2=\sqrt{\varepsilon_{\perp 2}-\sin^2\theta_i}\,,\qquad q_S=\sqrt{\varepsilon_S-\sin^2\theta_i}$$
$$A=(\omega a/c),\qquad L=(\omega l/c)$$

$$M=\begin{pmatrix} 1 & -1 & -1 & 0 & 0 & 0 \\ \cos\theta_i & q_1 & -q_1 & 0 & 0 & 0 \\ 0 & e^{iq_1A} & e^{-iq_1A} & -e^{iq_2A} & -e^{-iq_2A} & 0 \\ 0 & -q_1e^{iq_1A} & q_1e^{-iq_1A} & q_2e^{iq_2A} & -q_2e^{-iq_2A} & 0 \\ 0 & 0 & 0 & e^{iq_2L} & e^{-iq_2L} & -e^{iq_SL} \\ 0 & 0 & 0 & -q_2e^{iq_2L} & q_2e^{-iq_2L} & q_Se^{iq_SL} \end{pmatrix}\qquad \mathbf{e}=\frac{1}{E_I}\begin{pmatrix} E_R \\ E_{1+} \\ E_{1-} \\ E_{2+} \\ E_{2-} \\ E_S \end{pmatrix}\qquad \mathbf{b}=\begin{pmatrix} -1 \\ \cos\theta_i \\ 0 \\ 0 \\ 0 \\ 0 \end{pmatrix}$$

Thus, the reflection coefficient can be calculated by inverting the system above: $\boxed{R_{TE}=\left|\mathbf{e}(1)\right|^2}$.

<u>TM polarization</u>. We assume a TM polarized field $\left(E_y=0\right)$:

$$\mathbf{E}(x,z,t)=\left[E_I\cos\theta_i e^{i\frac{\omega}{c}\cos\theta_i z}+E_R\cos\theta_i e^{-i\frac{\omega}{c}\cos\theta_i z}\right]e^{i\frac{\omega}{c}\sin\theta_i x-i\omega t}\hat{x}+\left[-E_I\sin\theta_i e^{i\frac{\omega}{c}\cos\theta_i z}+E_R\sin\theta_i e^{-i\frac{\omega}{c}\cos\theta_i z}\right]e^{i\frac{\omega}{c}\sin\theta_i x-i\omega t}\hat{z}$$
$$\mathbf{H}(x,z,t)=\frac{1}{\mu_0 c}\left[E_I e^{i\frac{\omega}{c}\cos\theta_i z}-E_R e^{-i\frac{\omega}{c}\cos\theta_i z}\right]e^{i\frac{\omega}{c}\sin\theta_i x-i\omega t}\hat{y}\qquad z<0$$

$$\mathbf{E}(x,z,t)=\left[E_{1+}e^{i\frac{\omega}{c}\sqrt{\varepsilon_{\perp 1}-\frac{\varepsilon_{\perp 1}}{\varepsilon_{z1}}\sin^2\theta_i}\,z}+E_{1-}e^{-i\frac{\omega}{c}\sqrt{\varepsilon_{\perp 1}-\frac{\varepsilon_{\perp 1}}{\varepsilon_{z1}}\sin^2\theta_i}\,z}\right]e^{i\frac{\omega}{c}\sin\theta_i x-i\omega t}\hat{x}+\frac{\varepsilon_{\perp 1}\sin\theta_i}{\varepsilon_{z1}\sqrt{\varepsilon_{\perp 1}-\frac{\varepsilon_{\perp 1}}{\varepsilon_{z1}}\sin^2\theta_i}}\left[-E_{1+}e^{i\frac{\omega}{c}\sqrt{\varepsilon_{\perp 1}-\frac{\varepsilon_{\perp 1}}{\varepsilon_{z1}}\sin^2\theta_i}\,z}+E_{1-}e^{-i\frac{\omega}{c}\sqrt{\varepsilon_{\perp 1}-\frac{\varepsilon_{\perp 1}}{\varepsilon_{z1}}\sin^2\theta_i}\,z}\right]e^{i\frac{\omega}{c}\sin\theta_i x-i\omega t}\hat{z}$$
$$\mathbf{H}(x,z,t)=\frac{1}{\mu_0 c}\frac{\varepsilon_{\perp 1}}{\sqrt{\varepsilon_{\perp 1}-\frac{\varepsilon_{\perp 1}}{\varepsilon_{z1}}\sin^2\theta_i}}\left[E_{1+}e^{i\frac{\omega}{c}\sqrt{\varepsilon_{\perp 1}-\frac{\varepsilon_{\perp 1}}{\varepsilon_{z1}}\sin^2\theta_i}\,z}-E_{1-}e^{-i\frac{\omega}{c}\sqrt{\varepsilon_{\perp 1}-\frac{\varepsilon_{\perp 1}}{\varepsilon_{z1}}\sin^2\theta_i}\,z}\right]e^{i\frac{\omega}{c}\sin\theta_i x-i\omega t}\hat{y}\qquad 0<z<a$$

$$\mathbf{E}(x,z,t)=\left[E_{2+}e^{i\frac{\omega}{c}\sqrt{\varepsilon_{\perp 2}-\frac{\varepsilon_{\perp 2}}{\varepsilon_{z2}}\sin^2\theta_i}\,z}+E_{2-}e^{-i\frac{\omega}{c}\sqrt{\varepsilon_{\perp 2}-\frac{\varepsilon_{\perp 2}}{\varepsilon_{z2}}\sin^2\theta_i}\,z}\right]e^{i\frac{\omega}{c}\sin\theta_i x-i\omega t}\hat{x}+\frac{\varepsilon_{\perp 2}\sin\theta_i}{\varepsilon_{z2}\sqrt{\varepsilon_{\perp 2}-\frac{\varepsilon_{\perp 2}}{\varepsilon_{z2}}\sin^2\theta_i}}\left[-E_{2+}e^{i\frac{\omega}{c}\sqrt{\varepsilon_{\perp 2}-\frac{\varepsilon_{\perp 2}}{\varepsilon_{z2}}\sin^2\theta_i}\,z}+E_{2-}e^{-i\frac{\omega}{c}\sqrt{\varepsilon_{\perp 2}-\frac{\varepsilon_{\perp 2}}{\varepsilon_{z2}}\sin^2\theta_i}\,z}\right]e^{i\frac{\omega}{c}\sin\theta_i x-i\omega t}\hat{z}$$
$$\mathbf{H}(x,z,t)=\frac{1}{\mu_0 c}\frac{\varepsilon_{\perp 2}}{\sqrt{\varepsilon_{\perp 2}-\frac{\varepsilon_{\perp 2}}{\varepsilon_{z2}}\sin^2\theta_i}}\left[E_{2+}e^{i\frac{\omega}{c}\sqrt{\varepsilon_{\perp 2}-\frac{\varepsilon_{\perp 2}}{\varepsilon_{z2}}\sin^2\theta_i}\,z}-E_{2-}e^{-i\frac{\omega}{c}\sqrt{\varepsilon_{\perp 2}-\frac{\varepsilon_{\perp 2}}{\varepsilon_{z2}}\sin^2\theta_i}\,z}\right]e^{i\frac{\omega}{c}\sin\theta_i x-i\omega t}\hat{y}\qquad a<z<l$$

$$\mathbf{E}(x,z,t)=\left[E_S\hat{x}-\frac{\sin\theta_i}{\sqrt{\varepsilon_S-\sin^2\theta_i}}E_S\hat{z}\right]e^{i\frac{\omega}{c}\sqrt{\varepsilon_S-\sin^2\theta_i}\,z}e^{i\frac{\omega}{c}\sin\theta_i x-i\omega t}$$
$$\mathbf{H}(x,z,t)=\frac{1}{\mu_0 c}\frac{\varepsilon_S}{\sqrt{\varepsilon_S-\sin^2\theta_i}}E_S e^{i\frac{\omega}{c}\sqrt{\varepsilon_S-\sin^2\theta_i}\,z}e^{i\frac{\omega}{c}\sin\theta_i x-i\omega t}\hat{y}\qquad z>l$$

Imposing boundary conditions for the continuity of the magnetic field, the tangential electric field, and the orthogonal displacement vector at all the interfaces one gets $\boxed{M\mathbf{e}=\mathbf{b}}$ where:

$$q_1=\sqrt{\varepsilon_{\perp 1}-\frac{\varepsilon_{\perp 1}}{\varepsilon_{z1}}\sin^2\theta_i},\qquad q_2=\sqrt{\varepsilon_{\perp 2}-\frac{\varepsilon_{\perp 2}}{\varepsilon_{z2}}\sin^2\theta_i},\qquad q_S=\sqrt{\varepsilon_S-\sin^2\theta_i}$$

$$A=(\omega a/c),\qquad L=(\omega l/c)$$

$$M=\begin{pmatrix} \cos\theta_i & -1 & -1 & 0 & 0 & 0 \\ q_1 & \varepsilon_{\perp 1} & -\varepsilon_{\perp 1} & 0 & 0 & 0 \\ 0 & e^{iq_1A} & e^{-iq_1A} & -e^{iq_2A} & -e^{-iq_2A} & 0 \\ 0 & q_2\varepsilon_{\perp 1}e^{iq_1A} & -q_2\varepsilon_{\perp 1}e^{-iq_1A} & -q_1\varepsilon_{\perp 2}e^{iq_2A} & q_1\varepsilon_{\perp 2}e^{-iq_2A} & 0 \\ 0 & 0 & 0 & e^{iq_2L} & e^{-iq_2L} & -e^{iq_SL} \\ 0 & 0 & 0 & q_S\varepsilon_{\perp 2}e^{iq_2L} & -q_S\varepsilon_{\perp 2}e^{-iq_2L} & -q_2\varepsilon_S e^{iq_SL} \end{pmatrix}\qquad \mathbf{e}=\frac{1}{E_I}\begin{pmatrix} E_R \\ E_{1+} \\ E_{1-} \\ E_{2+} \\ E_{2-} \\ E_S \end{pmatrix}\qquad \mathbf{b}=\begin{pmatrix} -\cos\theta_i \\ q_1 \\ 0 \\ 0 \\ 0 \\ 0 \end{pmatrix}$$

Thus, also in this case, the reflection coefficient can be calculated by inverting the system above: $\boxed{R_{TM}=|\mathbf{e}(1)|^2}$.
